# Differentiating central nervous system infection from disease infiltration in hematological malignancy

**DOI:** 10.1038/s41598-022-19769-2

**Published:** 2022-09-22

**Authors:** Emma A. Lim, James K. Ruffle, Roshina Gnanadurai, Heather Lee, Michelle Escobedo-Cousin, Emma Wall, Kate Cwynarski, Robert S. Heyderman, Robert F. Miller, Harpreet Hyare

**Affiliations:** 1 Lysholm Department of Neuroradiology, University College London Hospitals NHS Foundation Trust, London, WC1N 3BG UK; 2 Department of Brain Repair and Rehabilitation, UCL Queen Square Institute of Neurology, London, WC1N 3BG UK; 3 Department of Infectious Disease, Hospital for Tropical Diseases & University College London Hospitals NHS Foundation Trust, London, NW1 2BU UK; 4 Department of Imaging, University College London Hospital, University College London Hospitals NHS Foundation Trust, London, NW1 2BU UK; 5 Department of Hematology, University College London Hospitals NHS Foundation Trust, London, NW1 2BU UK; 6 Division of Infection and Immunity, Research Department of Infection, UCL, London, WC1E 6JF UK

**Keywords:** Haematological diseases, Diseases of the nervous system, Central nervous system infections, CNS cancer, Neurology

## Abstract

Hematological malignancies place individuals at risk of CNS involvement from their hematological disease and opportunistic intracranial infection secondary to disease-/treatment-associated immunosuppression. Differentiating CNS infection from hematological disease infiltration in these patients is valuable but often challenging. We sought to determine if statistical models might aid discrimination between these processes. Neuroradiology, clinical and laboratory data for patients with hematological malignancy at our institution between 2007 and 2017 were retrieved. MRI were deep-phenotyped across anatomical distribution, presence of pathological enhancement, diffusion restriction and hemorrhage and statistically modelled with Bayesian-directed probability networks and multivariate logistic regression. 109 patients were studied. Irrespective of a diagnosis of CNS infection or hematological disease, the commonest anatomical distributions of abnormality were multifocal-parenchymal (34.9%), focal-parenchymal (29.4%) and leptomeningeal (11.9%). Pathological enhancement was the most frequently observed abnormality (46.8%), followed by hemorrhage (22.9%) and restricted diffusion (19.3%). Logistic regression could differentiate CNS infection from hematological disease infiltration with an AUC of 0.85 where, with OR > 1 favoring CNS infection and < 1 favoring CNS hematological disease, significantly predictive imaging features were hemorrhage (OR 24.61, *p* = 0.02), pathological enhancement (OR 0.17, *p* = 0.04) and an extra-axial location (OR 0.06, *p* = 0.05). In conclusion, CNS infection and hematological disease are heterogeneous entities with overlapping radiological appearances but a multivariate interaction of MR imaging features may assist in distinguishing them.

## Introduction

Hematological malignancies are a heterogeneous group of cancers that affect the blood, bone marrow and lymph nodes, and are usually classified into various types of lymphomas, leukemias, bone marrow failure and plasma cell disorders. Cumulatively, these conditions represent an increasing global healthcare burden. Hematological malignancies confer an increased risk of central nervous system (CNS) infection from an array of risk factors, including from disease-related disruption of immune function or the blood–brain barrier, as well as therapeutic interventions such as high dose chemotherapy, allogeneic hematopoietic stem cell transplantation (allo-HSCT) or chimeric antigen receptor T-cell therapy^1^.

CNS infection is associated with significant morbidity and mortality^2^, necessitating an accurate and timely diagnosis to enable prompt treatment. In the setting of allo-HSCT, for example, a variable incidence of between 5 and 15% has been reported. Despite this group being a significant ‘at-risk’ patient population for opportunistic CNS infection^3–5^, to date only limited data exists, predominantly focused on specific infectious agents or particular treatment regimens^5–10^. A wide range of imaging appearances for infection have been described^11^.

A dysregulated host immune response in systemic hematological malignancy can yield imaging findings in CNS infection which deviate from the classical features described in the literature. In these cases, the anticipated enhancement, perilesional oedema, and diffusion characteristics may be altered, weak or absent entirely^12,13^. Furthermore, the propensity of hematological malignancies to infiltrate the CNS can further confound image interpretation, with certain subtypes of lymphoma having an increased risk of CNS disease, such as non-Hodgkin lymphoma^14^. Hematological CNS disease dissemination is associated with poor outcomes^6,14,15^, and an accurate diagnosis is vital. Similar to infection^11^, the manifestations of intracranial hematological disease are heterogeneous and inclusive of parenchymal mass lesions, white matter abnormalities, ependymal, meningeal and bony abnormalities^14,16–19^. Comparing singular imaging features is often insufficient in discriminating between CNS infection and hematological disease infiltration. As such, a multi-faceted approach to reaching the diagnosis is plausibly required.

Given that both CNS infection and hematological disease infiltration are associated with reduced survival and necessitate divergent management pathways, the role of the radiologist in differentiating these two disease states is crucial. At odds with this need for diagnostic accuracy is the minimal available evidence to guide imaging interpretation in this context^5^. Therefore, there is a need for an approach that studies the diverse imaging appearances of these patients. We argue this is best characterized by sufficiently complex models that capture the major interactions between imaging features, which we establish in this article using a cohort of hematological oncology patients from a major tertiary hematology center.

## Methods

### Study population

We retrospectively identified adult and adolescent patients with hematological disease at our tertiary hematology center who underwent an MRI head study between October 2007 and September 2017, using an automated search of the medical electronic radiology information system for MRI head imaging requests made by the hematology department. Departmental approval was obtained for the retrospective collection of anonymous data, therefore written informed consent was not required. The search terms used for clinical data request were as follows: lymphoma, diffuse large B cell lymphoma (DLBCL), Waldenstrom’s Macroglobulinemia (WM), lymphoplasmacytic, Bing Neel, leukemia, acute lymphoblastic leukemia (ALL), acute myeloid leukemia (AML), chronic lymphocytic leukemia (CLL), chronic myeloid leukemia (CML), acute promyelocytic leukemia (APML), multiple myeloma, polyneuropathy organomegaly endocrinopathy myeloma and skin changes (POEMS) syndrome, and CNS infection (infection, infectious, infective, abscess, empyema, meningitis, encephalitis, meningoencephalitis, viral, bacterial, cytomegalovirus (CMV), Ebstein–Barr virus (EBV), herpes simplex virus (HSV), progressive multifocal leukoencephalopathy (PML), JC virus, human herpesvirus (HHV), HHV-6, varicella zoster virus (VZV), toxoplasmosis, cryptococcus, cryptococcal, tuberculosis (TB), cysticercosis, fungal, aspergillus, candida and mucormycosis). Search terms included all recognized synonyms and common misspellings. These data were collated and verified by a senior infectious disease clinician with over 30 years’ experience (RFM) using an electronic keyword search, and manually reviewed by a radiology resident. Exclusion criteria included patients without a diagnosis of hematological malignancy, patients with a negative keyword search, and any second or subsequent MRI Head study performed in each patient. A study consort flow diagram is reviewable as Supplementary Fig. 1.

Patient data was curated as part of their routine clinical care. The rationale for this was twofold. Firstly, it would facilitate research into this patient group with no interruption to their care, additional risks, or incurred treatment costs. Secondly, such a study would enable evaluation of the range of investigations—radiological, serological, or otherwise—that this type of cohort typically undergo during routine clinical care, as evidenced in detail elsewhere, see Refs.^20–24^.

Each MRI head study was independently reviewed by three separate individuals: an attending neuroradiologist and two radiology residents. The presence or absence of an imaging abnormality in one or more of six anatomical distributions was recorded electronically: (i) focal parenchymal, (ii) multifocal parenchymal, (iii) ependymal, (iv) leptomeningeal, (v) pachymeningeal and (vi) extra-axial. The presence of three additional radiological features: (i) pathological intracranial contrast enhancement on T1-weighted images following gadolinium-based intravenous contrast administration, (ii) diffusion restriction on diffusion weighted imaging and (iii) signal abnormalities on one or more sequences consistent with intracranial hemorrhage, was also recorded. Patient demographic data and the underlying hematological disease were obtained through retrospective interrogation of electronic patient records. Any discordance in recorded imaging data were reviewed with the senior attending neuroradiologist and a consensus reached.

Clinical and laboratory data for each patient were collected from electronic patient records, including (i) type of hematological malignancy, (ii) type of treatment (chemotherapy only vs. stem cell transplant), (iii) the presence of peripheral blood neutropenia or lymphopenia, (iv) cerebrospinal fluid analysis (hematology, biochemistry, culture, PCR, cytology) and (iv) each patient’s final clinical diagnosis, which represented the final decision of a multidisciplinary team meeting (MDT) based on a combination of the clinical, radiological, laboratory tests and pathological information. Patients were subsequently categorized as “CNS infection”, “CNS hematological disease” or “other”. Where CNS infection was diagnosed, the causative organism was recorded.

### Statistical analysis

#### Graphical analysis

A Bayesian graphical analysis was used to model the high dimensional interplay between imaging and other investigatory features in the formulation of a network^25–30^. In brief, this approach permits the allocation of patient imaging and clinical factors as individual ‘nodes’, and the commonality between them as ‘edges’. This approach yields a network that may reveal underlying structure to better understand the complex imaging appearances across the two heterogenous conditions. We undertook a systematic review to the use of ‘graph theory’ AND ‘central nervous system infection’ OR ‘central nervous system hematological disease’ (with all possible synonyms) and found no prior research in this domain to have taken this approach. The main aim of this approach was to facilitate modelling of complex interaction patterns between arrays of patient features, including a means to harmonize data across the imaging, other investigatory and clinical domains. All imaging, serological, cerebrospinal fluid and other diagnostic features were used as nodes of the network, and the strength of connection, or edges, between them derived by their conditional probability, P(X|Y) (Supplementary Fig. 2). For example, the probability of the cerebrospinal fluid (CSF) showing an abnormal finding, given the presence of an abnormal white cell count (WCC) on the CSF [P(CSF Abnormality|CSF WCC Abnormality)], must always equal one, since all abnormal CSF WCC would lead to an abnormal overall CSF result. Conversely, the probability of a CSF WCC abnormality, given the CSF has any abnormal finding [P(CSF WCC Abnormality|CSF Abnormality)] must lie in the range space of 0–1, since not all abnormal CSF findings are secondary to a deranged WCC. Bayes’ theorem is detailed elsewhere and we would recommend the following for further reading^31^. This process was computed for all possible pairwise features creating a directed graphical network, consisting of 21 nodes (the individual features) and 420 edges (binomial coefficient of $$\left(\begin{array}{c}21\\ 2\end{array}\right)$$, or ‘21 choose 2’, with both edge directions implicitly possible). Edges were weighted by the probabilistic occurrence of if one event was conditionally associated with another as described above. Directed edges with a probability of occurring of 0—i.e., those that were probabilistically implausible—were removed. After model fitting, the eigenvector centrality of features was ascertained, weighted by the directional conditional probability of the connecting edge. Eigenvector centrality provides an insight as to the ‘influence’ of a given imaging or clinical feature on the overall disease network^32^, and hence is a valuable approach to establish especially important features that may predict one diagnosis over another whilst still being appropriately modelled in a multivariate domain that accounts for the heterogeneity of the disease. Analysis was undertaken with graph-tool (https://graph-tool.skewed.de)^33^ in Python version 3.6. Graph layout visualization was computed by the scalable force directed placement (SFDP) spring block layout, weighted by the conditional probability of the edge and the total degree count of each feature (the number of connections to and from it).

#### Logistic regression

Multivariate logistic regression models were used to ascertain imaging features key to the differential diagnosis in the study group, emulating the heuristic of a radiologist reviewing the imaging. Four separate sequential models were constructed: first, a general abnormality detection model to identify features indicative of either CNS hematological disease or CNS infection; second, separate models to identify CNS infection in those proven positive from the remainder of the cohort; third, a separate model to identify CNS hematological disease from the remainder; and finally, a model to discriminate between both CNS hematological disease and CNS infection. All models used the binomial logit link, and variable selection was performed with stepwise feature selection with maximizing goodness of fit by the Akaike information criterion (AIC)^34^. All models were constructed using R (version 4.1.2), and for each the odds ratios for statistically significant (p < 0.05) features were extracted and the area under the receiver operator characteristic curve (ROC) computed.

### Conference presentation

Preliminary data of this work were presented in oral format at the 2021 European Congress of Radiology.

## Results

### Patient demographics

**Figure 1:**
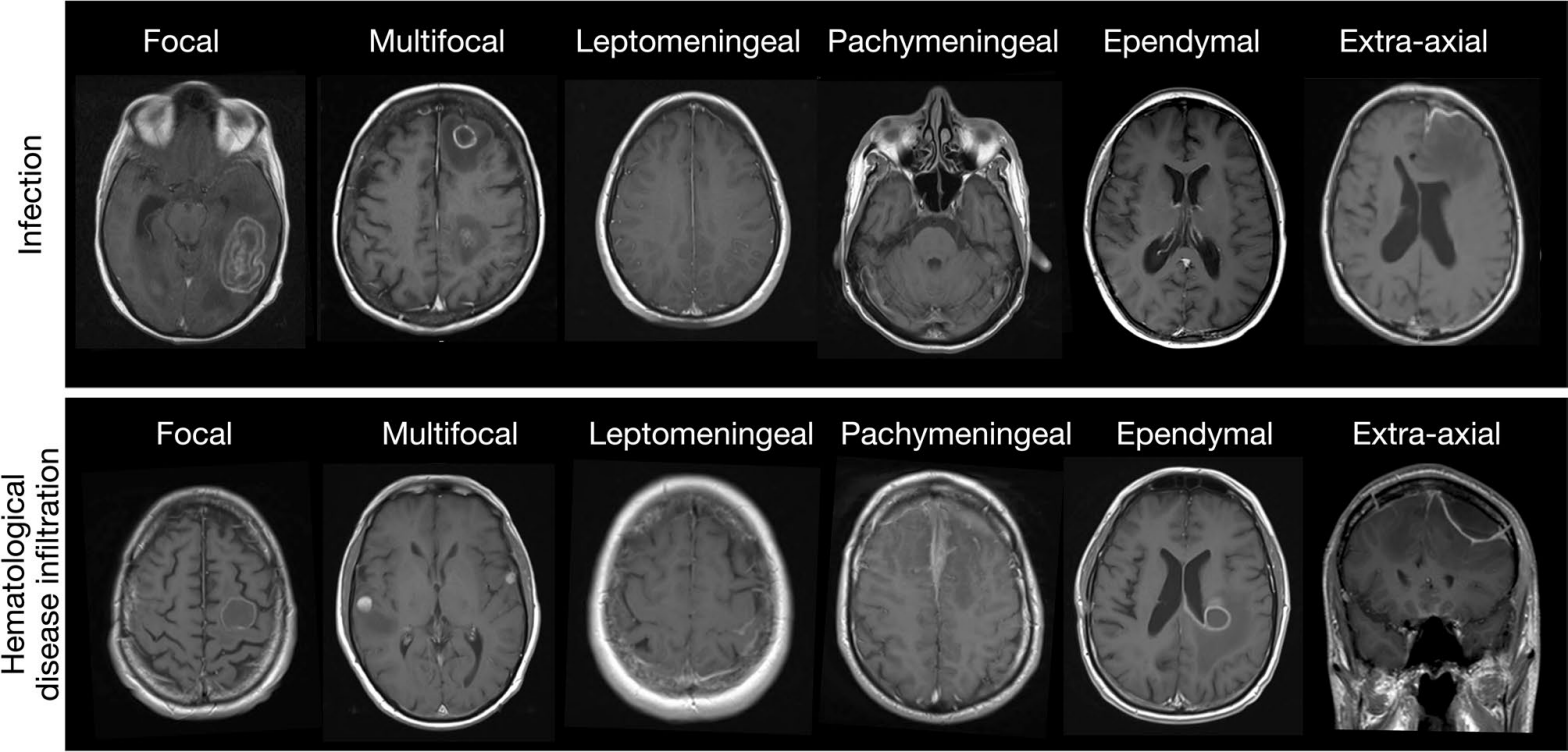
Case examples of anatomical imaging classification in MRI head studies with confirmed final diagnosis of either CNS infection of hematological disease involvement.

1855 MRI head attendances were screened for eligibility, with 1746 excluded as the second or subsequent study for any individual (n = 488) or having returned a negative key term search for both an underlying hematological malignancy and suspected infection (n = 1258) (Fig. 1). The remaining 109 patients formed our study sample [median age 52 years (range 13–81); 56 (51.4%) male; 94 (86.2%) inpatient] (Table 1). The recorded underlying hematological diagnoses were lymphoma (n = 50), leukemia (n = 51), myeloma (n = 4), myelodysplastic syndrome (n = 3) and monoclonal gammopathy of uncertain significance (n = 1). All patients had received standard chemotherapy to treat their underlying malignancy. 27 patients had received stem cell transplantation, from which 23 were allogeneic stem cell transplants, and 4 were autologous stem cell transplants. CSF data were available in 53 patients (48.6%); of which 36 (67.9%) demonstrated an abnormality [protein (n = 21), PCR positivity (n = 13), WCC (n = 11), blasts (n = 6), bacterial growth (n = 3), glucose. (n = 2) and India Ink stain positivity (n = 1)]. Neutropenic and lymphopenic status were available in 81 of 109 patients. Peripheral blood neutropenia 1 month surrounding the MRI scan was observed in 35 of 81 patients (43.2%) and peripheral blood lymphopenia in 34 of the 81 patients (41.9%).

**Table 1:** Cohort demographics.

Feature	Value
Age (range)	52 (13–81)
Gender	56 male, 53 female
Diagnosis	Lymphoma (n = 50), leukemia (n = 51), myeloma (n = 4), myelodysplastic syndrome (n = 3) and monocloncal gammopathy of uncertain significance (n = 1)
Complication	CNS infection (n = 28), CNS hematological disease dissemination (n = 15)

The 28 patients (25.7%) diagnosed with intracranial infection [median age 55 years (range 15–81); 16 (57.1%) male] had an underlying diagnosis of leukemia (n = 12), lymphoma (n = 14), myeloma (n = 1) and myelodysplastic syndrome (n = 1). A range of CNS infections were diagnosed: cytomegalovirus (n = 6) and other viral encephalitides (HSV, HHV6, unspecified) (n = 4), pseudomonas aeruginosa (n = 3) and enterovirus meningitis (n = 1), unspecified pyogenic abscess (n = 1), fungal [(n = 4), aspergillosis (n = 3), cryptococcus (n = 1), saccharomyces (n = 1), parasitic (toxoplasmosis n = 4) and JC virus (n = 3)]. The 15 patients (13.8%) diagnosed with CNS involvement of their hematological malignancy (median age 56 years (range 19–72); (8 (53.3%) male) had an underlying diagnosis of lymphoma (n = 10, including 1 case of Waldenstrom macroglobulinemia) and leukemia (n = 5). 66 patients (60.6%) received an alternative diagnosis other than CNS infection or CNS involvement of their hematological malignancy. Based upon the final MDT outcomes, radiological deep phenotyped data were segregated into (i) CNS infection (n = 28), (ii) CNS hematological disease (n = 15) and (iii) other treatment-related changes, previous infarction, cerebral microangiopathy, microhemorrhage, subdural hematoma or hygroma, pachymeningeal abnormalities secondary to lumbar puncture (LP), or a normal intracranial appearance (n = 10).

### Abnormal diagnostic investigations in patients with hematological malignancy

#### Frequency of abnormalities

Within our sample, the commonest abnormalities were localized to multifocal parenchymal (n = 38; 34.9%), focal parenchymal (n = 32; 29.4%), leptomeningeal (n = 13; 11.9%), dural (n = 12; 11%), extra-axial (n = 8; 7.3%) and ependymal (n = 7; 6.4%) (Fig. 1). Pathological enhancement was the most frequently observed abnormality (n = 51; 46.8%), followed by hemorrhage (n = 25; 22.9%) and restricted diffusion (n = 21; 19.3%). The network coalescing imaging features, blood tests and LP findings for all patients demonstrating the complex interplay between these features across complimentary domains is further discussed in the supplementary material and visualized in Supplementary Fig. 2.

### Diagnostic features of CNS hematological disease infiltration

#### Frequency of abnormalities

**Figure 2:**
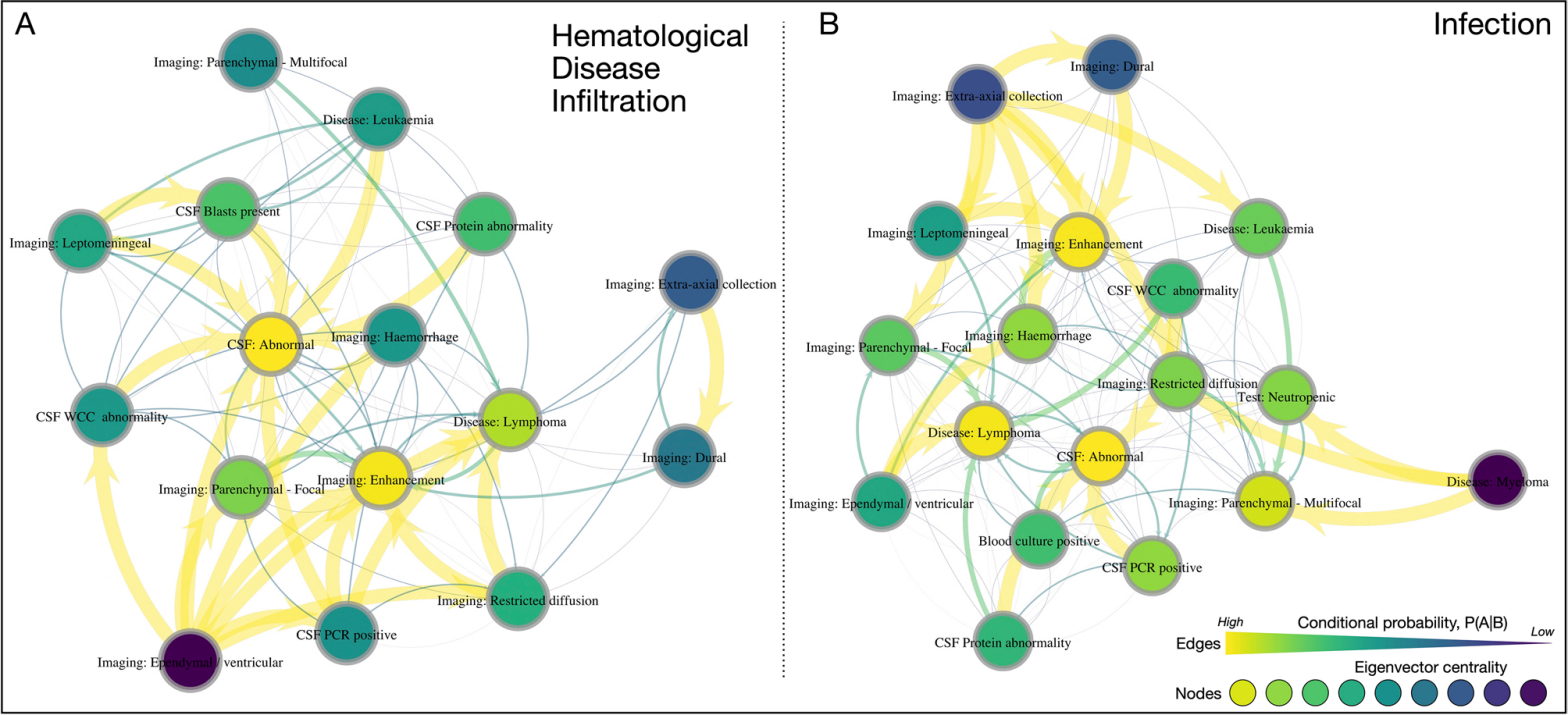
Visual network analysis of CNS hematological disease involvement and infection. (**A**) Graphical representation of CNS hematological disease feature network. (**B**) Graphical representation of CNS infection feature network. Nodes are color-coded according to their eigenvector centrality, and edge size and color is proportional to the directed conditional probability, with color key as shown. *CSF* cerebrospinal fluid, *PCR* polymerase chain reaction, *WCC* white cell count.

In the CNS hematological disease infiltration subgroup, the abnormalities were localized to focal parenchymal (n = 7; 46.7%), multifocal parenchymal (n = 5; 33.3%), dural (n = 4; 26.7%), leptomeningeal (n = 4; 26.7%), extra-axial (n = 3; 20%) and ependymal (n = 1; 6.7%). Pathological enhancement was the most frequently observed abnormality (n = 12; 80%), followed by restricted diffusion (n = 5; 33%) and hemorrhage (n = 3; 20%). Construction of the directed graphical network of features in the hematological disease CNS involvement subgroup yielded a 19-node network of 196 edges (Fig. 2A). There was a high degree of heterogeneity amongst this subgroup, yet there were several highly probable directed links between imaging and other investigatory features. For example, ependymal or ventricular abnormalities were highly associated with several multi-modal features, ranging from abnormal diffusion, focal parenchymal abnormalities, hemorrhage, a PCR-positive or abnormal CSF WCC on LP, which in turn were well associated with an overall abnormal CSF sample, a diagnosis of lymphoma and abnormal intracranial enhancement.

### Diagnostic features of CNS infection in patients with hematological malignancy

#### Frequency of abnormalities

In the infection subgroup, the abnormalities were localized to multifocal parenchymal (n = 16; 57.1%), focal parenchymal (n = 7; 25%), leptomeningeal (n = 4; 14.3%), ependymal (n = 4; 14.3%), dural (n = 2; 7.1%) and extra-axial (n = 1; 3.6%). Pathological enhancement was the most frequently observed abnormality (n = 15; 53.6%), followed by restricted diffusion (n = 9; 32.1%) and hemorrhage (n = 9; 32.1%). Construction of the directed graphical network of features in the CNS infection subgroup yielded a 19-node network of 232 edges, containing more possible edges than the CNS hematological disease counterpart (Fig. 2B). This process appeared to identify a segregation of features into two broad feature groups linked by the imaging features of intracranial hemorrhage, extra-axial abnormality, and focal parenchymal abnormality. The former coalesced the presence of dural abnormalities, extra-axial collections, leptomeningeal, focal parenchymal and pathological enhancement, whilst the latter included CSF and blood culture findings and multifocal parenchymal abnormalities. Diffusion abnormality appeared to link these two feature groups. The features with greatest eigenvector centrality were, again, abnormal enhancement, an abnormal CSF result, a known history of lymphoma, followed by a range of discrete imaging, serological, or CSF tests.

### Comparison of diagnostic investigations in CNS infection and hematological disease involvement subgroups

**Figure 3:**
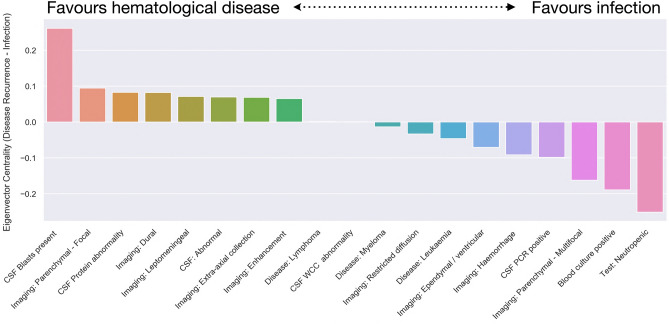
Quantitative graphical centrality differences of diagnostic features between CNS hematological disease involvement and infection. Absolute difference in the normalized eigenvector centrality of features, weighted by directed conditional probability. Features with value above zero (left side of bar plots) favor the CNS hematological disease involvement group and features with value below zero favor the CNS infection group (right side of bar plots). *CSF* cerebrospinal fluid, *PCR* polymerase chain reaction, *WCC* white cell count.

Having derived the eigenvector centrality measures from the imaging, serological and CSF features, cross comparison between the CNS hematological disease and CNS infection subgroups was performed (Fig. 3). This process identified key features more ‘influential’ in the pathology network for each group. Features that favored hematological disease involvement were the presence of blasts in CSF, in addition to focal parenchymal abnormalities, CSF protein abnormality, dural, leptomeningeal or pathological enhancement abnormalities and extra-axial abnormalities. In contrast, features that favored CNS infection were the presence of neutropenia, in addition to a positive blood culture, multifocal parenchymal abnormalities, a positive CSF PCR, intracranial hemorrhage, ependymal or ventricular abnormality, restricted diffusion and, albeit weakly, an underlying diagnosis of either myeloma or leukemia.

### Predicting diagnosis by imaging features

**Figure 4:**
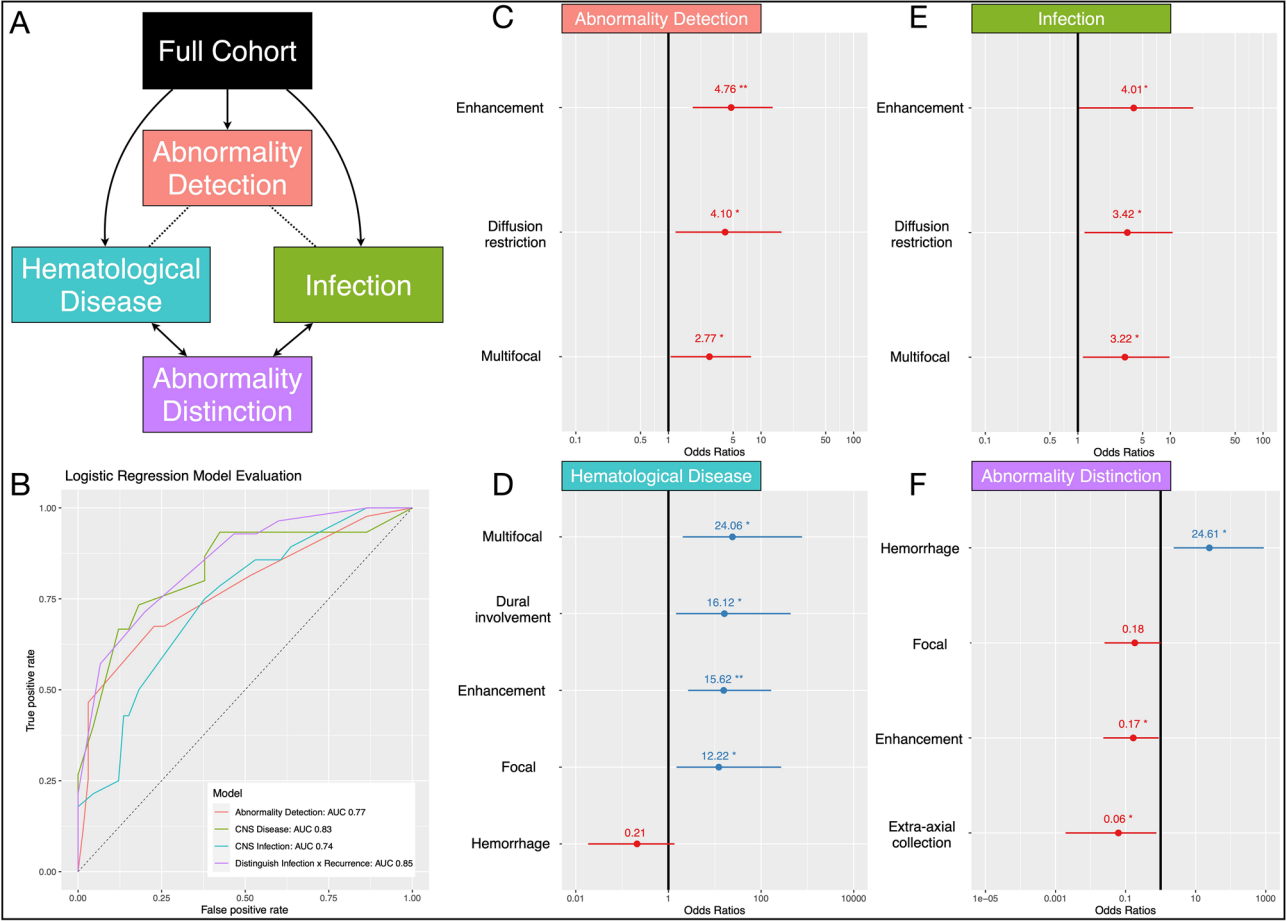
Modelling to identify and differentiate CNS hematological disease involvement and infection with imaging features alone. (**A**) Models were constructed with the aim to emulate the cognitive heuristic of a reading radiologist, wherein a first layer model of abnormality detection is derived, in this case identifying either of CNS hematological disease involvement or infection (red box). Following which, separate models to aid in the differential of either CNS hematological disease involvement (teal box) or infection (green box) are constructed to identify predictive features. Lastly, a model in effort to differentiate the two is built (purple box). (**B**) Receiver operator characteristic (ROC) plots of these four models, color coded as above. Beneficial features and Odds ratios with 95% confidence intervals for each model are shown on a log scale in (**C**–**F**). (**C**) Abnormality detection model identified three favoring features. (**D**) The CNS hematological disease involvement model identified 4 features favoring it (OR > 1) and one that made it less likely (OR < 1). (**E**) The CNS infection model identified 3 features favoring it. (**F**) The abnormality distinction model identified 1 feature favoring CNS infection (OR > 1) and 3 features favoring CNS involvement of the hematological disease (OR < 1). Asterisks correspond to p values as per conventional standards: **p < 0.01,*p < 0.05. *CSF* cerebrospinal fluid, *PCR* polymerase chain reaction, *WCC* white cell count.

Multivariate logistic regression models were fitted to determine if specific arrays of imaging features held some predictive value over ascertaining either diagnosis. These were constructed in a formulaic manner with an intent to emulate the cognitive heuristics used by a radiologist, wherein firstly an abnormality is either identified or not in this cohort, which could be *either* due to CNS infection or CNS hematological disease, following which an attempt is made to discriminate between the two differentials (Fig. 4).

#### Abnormality detection

The abnormality detection logistic regression model identified three imaging features significantly related to the identification of general abnormality which might be either secondary to infection or CNS disease. These were abnormal intracranial enhancement [Odds Ratio (OR) 4.76, 95% CI 1.78–12.76, *p* = 0.002], restricted diffusion (OR 4.10, 95% CI 1.13–14.88, *p* = 0.03), and multifocal parenchymal abnormality (OR 2.77, 95% CI 1.03–7.47, *p* = 0.04). This model achieved an area under the curve (AUC) of 0.77.

#### CNS hematological disease prediction

The hematological disease involvement model identified 4 significant features in identifying hematological CNS disease from the remainder of the cohort. These were multifocal parenchymal abnormality (OR 24.06, 95% CI 1.44–403.22, *p* = 0.02), dural abnormality (OR 16.12, 95% CI 1.17–232.65, *p* = 0.04), intracranial enhancement (OR 15.62, 95% CI 2.14–113.92, *p* = 0.007) and focal parenchymal abnormality (OR 12.22, 95% CI 1.12–133.51, *p* = 0.04). A trend in utility for the *absence* of hemorrhage to identify CNS hematological disease was apparent. Whilst non-significant, this was retained by the automated stepwise feature selection model in optimization as per the minimum AIC (OR 0.21, 95% CI 0.027–1.66, *p* = 0.13). This model achieved an AUC of 0.83.

#### CNS infection prediction

The CNS infection model identified 3 significant features in identifying CNS infection from the remainder of the cohort. These were intracranial enhancement (OR 3.42, 95% CI 1.16–10.10, *p* = 0.02), multifocal parenchymal abnormality (OR 3.22, 95% CI 1.11–9.33, *p* = 0.03) and restricted diffusion (OR 4.01, 95% CI 1.00–16.13, *p* = 0.05). This model achieved an AUC of 0.74.

#### Distinguishing CNS infection from CNS hematological disease

**Figure 5:**
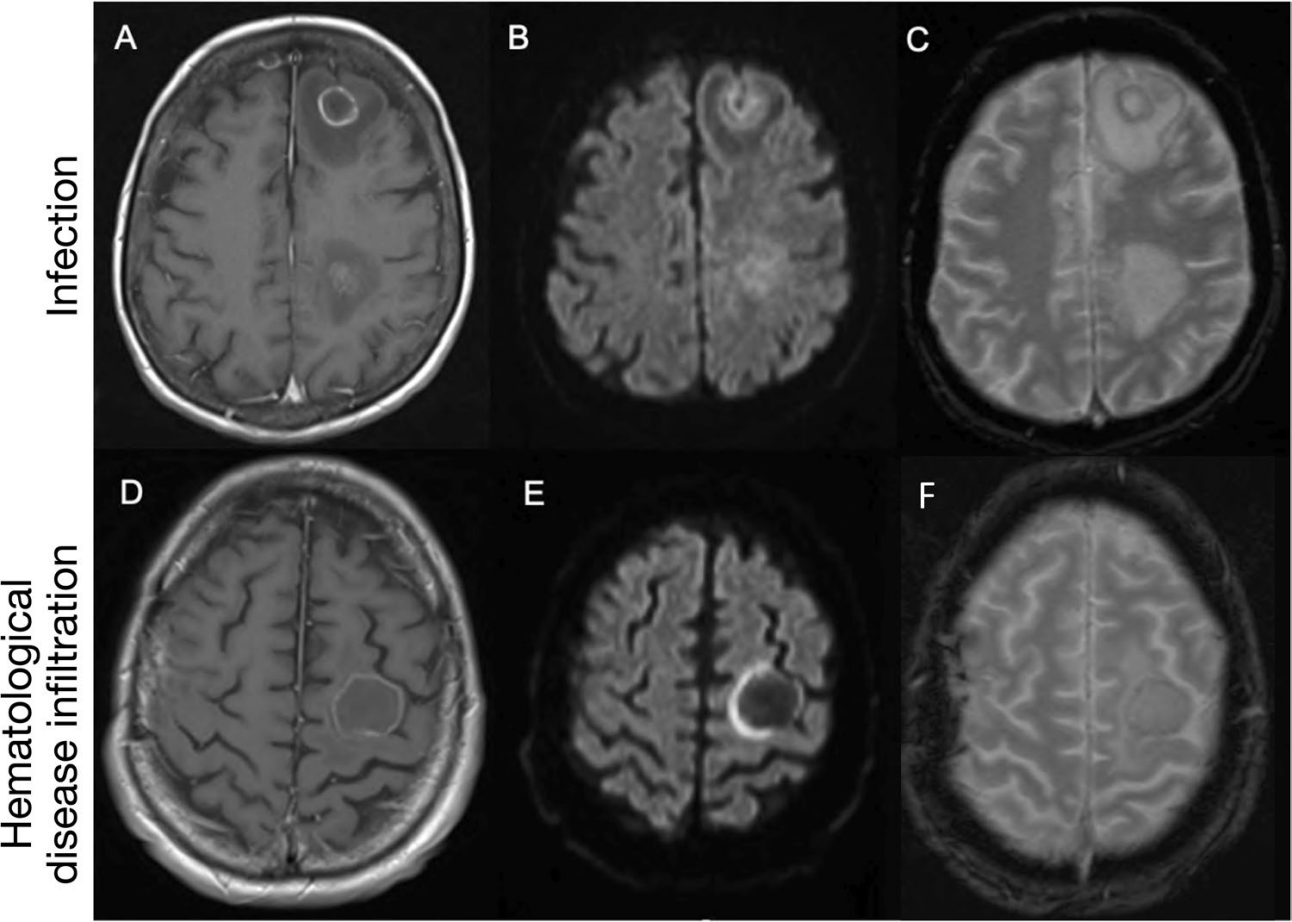
Case example of multivariate abnormality differentiation model. Cerebral aspergillosis in a patient with chronic lymphocytic leukemia (**A**–**C**) and secondary CNS lymphoma in a patient with DLBCL (**D**–**F**). Rim enhancing parenchymal lesions are demonstrated within the left frontal lobe in both cases (**A**,**D**) with peripheral restricted diffusion (**B**,**E**). The former is multifocal in distribution, whereas the latter is solitary. Peripheral hypointensity on T2* GRE (**C**) in the patient with cerebral aspergillosis is keeping with prior hemorrhage, whereas no evidence of hemorrhage was demonstrated in the case of DLBCL (not shown). *CNS* central nervous system, *DLBCL* diffuse large B-cell lymphoma.

A logistic regression model constructed to differentiate CNS infection from hematological disease involvement identified 4 features beneficial in doing so. With OR > 1 favoring infection, and < 1 favoring CNS disease, these were: hemorrhage (OR 24.61, 95% CI 1.48–409.02, *p* = 0.02), focal parenchymal abnormality [retained in minimizing the AIC, albeit non-significant] (OR 0.18, 95% CI 0.03–1.11, *p* = 0.06), enhancement (OR 0.17, 95% CI 1.48–409.01, *p* = 0.04), and extra-axial (OR 0.06, 95% CI 0.00–0.99, *p* = 0.05). This model achieved an AUC of 0.85 in fitting. We provide a case-based example of the model heuristic as Fig. 5.

## Discussion

Here, we quantify the value of MRI in the diagnosis of, and differentiation between, CNS infection and CNS hematological disease infiltration when treated in a multivariate domain that facilitates the interaction between imaging features. We reveal the phenotypic networks that plausibly underpin the heterogenous intracranial appearances and investigatory findings of both intracranial infection and CNS disease manifestations in patients with hematological malignancy in a large tertiary referral center. These findings illustrate that, whilst singular imaging features may be of variable benefit in discriminating the conditions, the harmony between the presence and/or absence of imaging findings in a multivariate space offers benefit in discriminating what can otherwise be two disorders that are classically—in the reporting room—hard to segregate. The utility of this modelled approach is demonstrated with companion cases in Fig. 5, illustrating that whilst singular imaging features seem relatively non-specific, a multivariate approach permitted segregation of the two diagnoses.

Reassuringly, both the clinical factors and radiological findings that were of statistically significant value in the identification of and differentiation between the two disease states were in keeping with the larger evidence base amassed for patients with generalized immunosuppression^5,7,11–13,35–37^, where one may infer similar alterations in disease behavior. Therefore, these results should give radiologists greater confidence in utilizing these features in this population and serve as an incremental step in furthering the understanding of these complex processes.

### Differential diagnosis in intracranial appearances

Imaging is frequently an important diagnostic test performed in a patient with prior hematological malignancy, where there is a high risk for opportunistic CNS infection or hematological disease involvement. MRI is perfectly suited as a non-invasive tool to help guide clinical management decisions, especially in cases where LP and biopsy are unobtainable. Whilst CSF analysis and brain biopsy remain the gold standard in the diagnosis of CNS infection and hematological disease involvement, they are invasive and associated with both false-positive and false-negative results^38^. As such, an effort to ascertain key imaging features that might act as indicators of a specific abnormality, or aid in differentiating the two, is justified. Here, we provide a series of preliminary models, first seeking to identify abnormalities which suggest either infection or hematological disease involvement, followed by a model to interrogate which of the two is more probable in an area of existing uncertainty. The findings illustrate imaging features which are likely to help guide this process.

### Imaging features

Regarding imaging features, the presence of intralesional hemorrhage was the most discriminatory. Hemorrhage can be seen in a variety of CNS infections but is infrequent in untreated CNS hematological disease^19^. In HSV-1 encephalitis, hemorrhage is of variable appearance depending on the chronicity of the blood products, although commonly presents as regions of T1-hyperintensity or GRE T2*/SWI hypointensity with ‘blooming’^39^. Peripheral hemorrhage can be seen in cerebral toxoplasmosis^40^ and aspergillus may be hemorrhagic^41^. The presence of restricted diffusion was not discriminatory as this finding is encountered in both CNS infection and CNS hematological disease infiltration. Although homogenous diffusion restriction in the central necrotic core is seen in pyogenic abscesses^42^, restricted diffusion of the lesion wall can be seen in toxoplasmosis^40^ and aspergillosis^41^, similar to that observed in CNS lymphoma in the immunosuppressed state. The presence of enhancement, a focal abnormality or an extra-axial abnormality were, to a lesser extent, more suggestive of CNS hematological disease involvement, rather than infection, in our cohort. There is potential bias in these findings as three of our CNS infection cases had a final diagnosis of viral encephalitis without pathological enhancement on MRI, whilst two patients with extra-axial abnormalities both had a final diagnosis of relapsed B-cell lymphoma. However, knowledge of these findings can aid the radiologist when assessing MR images and taken together with clinical information, can improve discrimination of CNS infection from CNS hematological disease.

### Strengths and limitations

Whilst the study appears to encompass a small sample size, this outcome was the result of a thorough search over a 10-year period at our tertiary level hematology referral center which receives referrals across a nationwide catchment area. It therefore seems appropriate to draw inference from this sample. This work is similar in size to the largest currently described in the literature, which focuses primarily on clinical guidance^5,37^. Due to the retrospective study design and limitations of the electronic patient records system, complete data regarding neurological examination findings, corticosteroid administration and timing, neutropenic status and bone marrow transplantation status was not achievable, and investigation of any features not captured in this article should be addressed in future work. To our strength, this is the largest cohort of its kind collected for this specific analysis and consisted of every hematological oncology patient treated at a major tertiary center over a ten-year period. Whilst the sample size may appear relatively small compared to the timeframe for collection, the adverse outcomes associated with CNS infection, CNS manifestations of hematological disease or the misdiagnosis of either of these confers a clinical importance that can be considered disproportionate to the number of patients affected. Future work should include prospective evaluation of the most discerning radiological parameters to provide external validation of their use in these contexts.

## Conclusions

We present a comprehensive imaging analysis of the largest, most studied group of individuals with hematological malignancy with either superadded CNS infection or disease dissemination. This is a complex disease group wherein exists much diagnostic uncertainty due to overlapping and variable imaging features. We provide a framework to better understand the disease systems that underpin this heterogeneity and identify important imaging features which, whilst largely uninformative taken as singular entities, when coalesced to multivariate models offer value in differentiating intracranial infection from CNS disease in patients with hematological malignancy. Further studies should validate and refine these models in additional, larger, patient cohorts.

## Supplementary Information

Supplementary Figure 1.

Supplementary Figure 2.

Supplementary Information.

## Data Availability

The datasets generated during and/or analyzed during the current study are not publicly available under the framework that governs its use. Modelling code however is available from the corresponding author on reasonable request.
