# Author Correction: Differentiating central nervous system infection from disease infiltration in hematological malignancy

**DOI:** 10.1038/s41598-022-25508-4

**Published:** 2022-12-05

**Authors:** Emma A. Lim, James K. Ruffle, Roshina Gnanadurai, Heather Lee, Michelle Escobedo-Cousin, Emma Wall, Kate Cwynarski, Robert S. Heyderman, Robert F. Miller, Harpreet Hyare

**Affiliations:** 1 Lysholm Department of Neuroradiology, University College London Hospitals NHS Foundation Trust, London, WC1N 3BG UK; 2 Department of Brain Repair and Rehabilitation, UCL Queen Square Institute of Neurology, London, WC1N 3BG UK; 3 Department of Infectious Disease, Hospital for Tropical Diseases and University College London Hospitals NHS Foundation Trust, London, NW1 2BU UK; 4 Department of Imaging, University College London Hospital, University College London Hospitals NHS Foundation Trust, London, NW1 2BU UK; 5 Department of Hematology, University College London Hospitals NHS Foundation Trust, London, NW1 2BU UK; 6 Division of Infection and Immunity, Research Department of Infection, UCL, London, WC1E 6JF UK

Correction to: *Scientific Reports* https://doi.org/10.1038/s41598-022-19769-2, published online 22 September 2022

The Acknowledgements section in the original version of this Article was incomplete.

“JR was supported by CDT i4health. HH is supported by the UCLH NIHR Biomedical Research Centre.”

now reads:

“JR was supported by CDT i4health, the Guarantors of Brain, the NHS Topol Digital Fellowship and the Medical Research Council (MR/X00046X/1). HH is supported by the UCLH NIHR Biomedical Research Centre.”

In addition, in the original version of this Article, James K. Ruffle was omitted as a corresponding author. Correspondence and requests for materials should also be addressed to j.ruffle@ucl.ac.uk.

The original Article has been corrected.

