# Supplementary figures and images for: Differentiating central nervous system infection from disease infiltration in hematological malignancy
Source: Sci Rep. 2022 Sep 22;12:15805. doi: 10.1038/s41598-022-19769-2 (PMC9499957; doi:10.1038/s41598-022-19769-2)

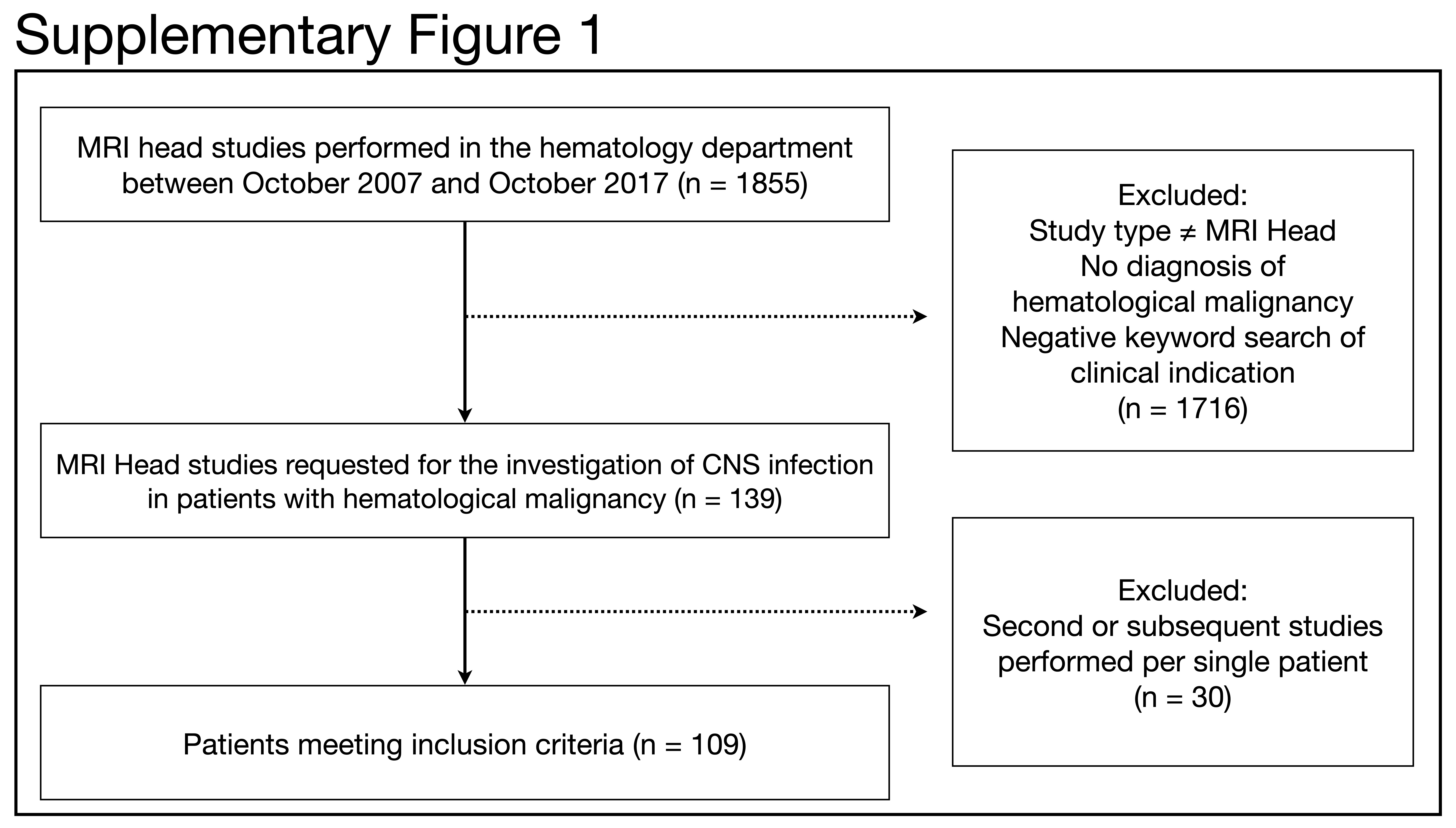

Supplement: Supplementary file 1 — Supplementary Figure 1. [file 41598_2022_19769_MOESM1_ESM.jpg]

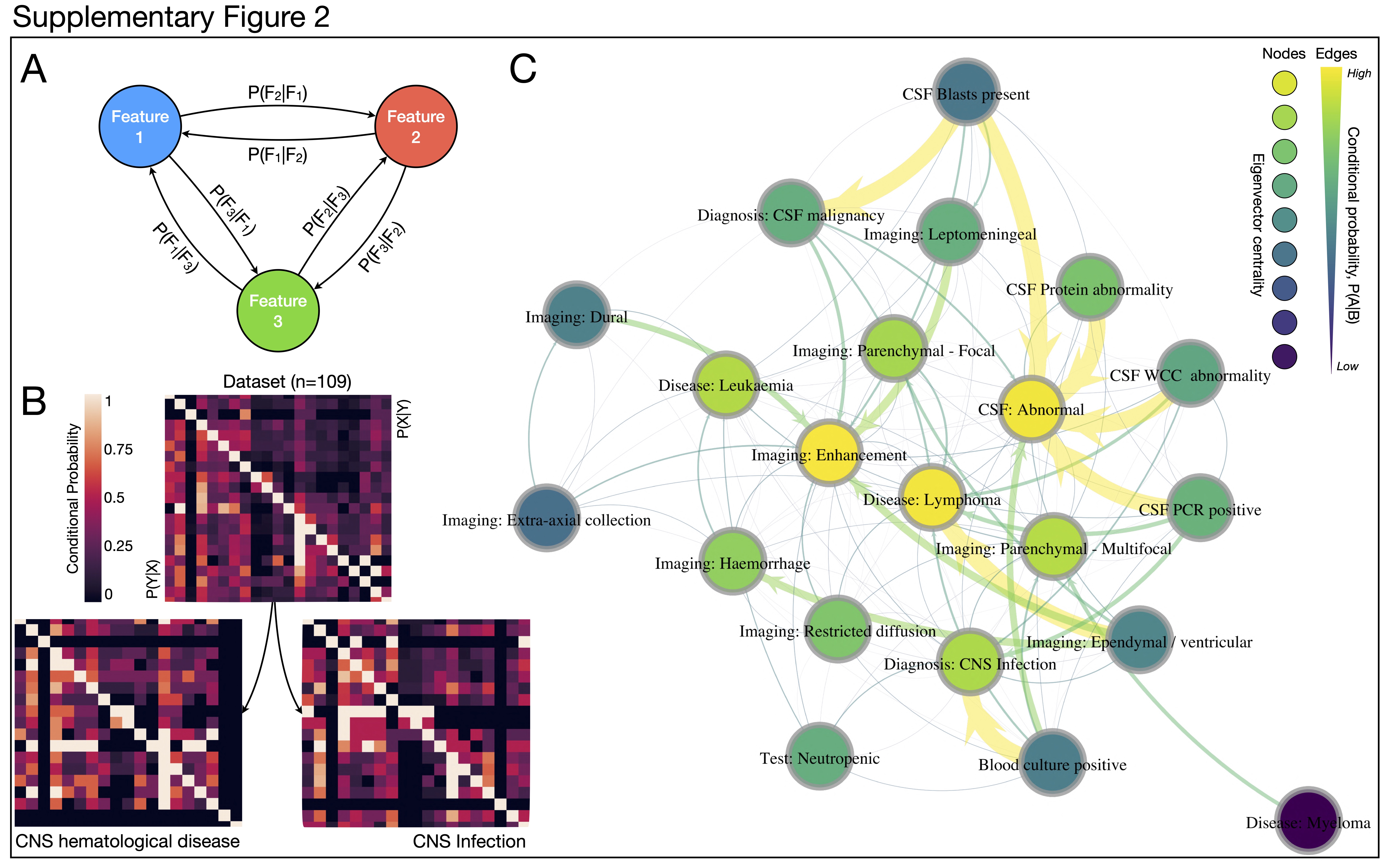

Supplement: Supplementary file 2 — Supplementary Figure 2. [file 41598_2022_19769_MOESM2_ESM.jpg]
