# Supplementary material for: Differentiating central nervous system infection from disease infiltration in hematological malignancy
Source: Sci Rep. 2022 Sep 22;12:15805. doi: 10.1038/s41598-022-19769-2 (PMC9499957; doi:10.1038/s41598-022-19769-2)
Supplement: Supplementary file 3 — Supplementary Information. [file 41598_2022_19769_MOESM3_ESM.docx]

# TITLE

Differentiating central nervous system infection from disease infiltration in hematological malignancy

# SUPPLEMENTARY MATERIAL

# SUPPLEMENTARY METHODS

**Image data acquisition**

MRI scans were obtained at 1.5T (Siemens Avanto) and 3T (Philips Achieva). Patients underwent routine MR Head with contrast protocol which included: T1-weighted, T2-weighted, FLAIR, Diffusion Weighted Imaging (DWI), heme-sensitive sequence (T2* or Susceptibility-Weighted Imaging (SWI) and T1-weighted post-contrast sequences. A small number of patients did not receive contrast enhancement due to poor renal function or a truncated study secondary to seizure, claustrophobia, or poor clinical status (n=7).

# SUPPLEMENTARY RESULTS

**Graphical representation of abnormalities**

This confirmed the heterogeneity of the group with regards to their possible radiological and other investigation findings, as plausible links were demonstrated between most features; 373 directed edges were present out of a possible 420 (in a 21-node network). Many links were unlikely given their low conditional probability values, but several edges linking imaging and other features exhibited reasonably high probability values. For example, within this dataset, ependymal or ventricular abnormalities were highly likely to be associated with lymphoma, specifically. The presence of ependymal/ventricular abnormalities were also frequently associated with additional imaging features, here abnormal enhancement, or hemorrhage. However, neither pathological enhancement nor hemorrhage were particularly predictive for a diagnosis of lymphoma. Rather, these features served as important links to other abnormalities, such as leptomeningeal or dural abnormalities being strongly linked to pathological enhancement.

This graphical representation of the data illustrates the hierarchical nature of the features. For instance, where a LP was completed, if blasts were present on the CSF sample, this would, as expected, conditionally link the diagnosis to be CNS hematological disease and that the CSF sample was abnormal. Additionally, this would also link to a higher-order pathway wherein a leptomeningeal abnormality on MRI would be more likely, which if positive, would also likely link to a focal parenchymal abnormality, which in turn would likely link to an abnormal focus of enhancement. We reviewed the eigenvector centrality of features, a putative measure which identifies those with greatest ‘influence’ on the structure of the whole dataset. This revealed that, irrespective of if the final diagnosis were infection or relapse, the presence of abnormal intracranial enhancement, an abnormal LP test and an underlying diagnosis of lymphoma, followed by the presence of hemorrhage, focal or multifocal parenchymal abnormalities, were those features were greatest centrality.

# SUPPLEMENTARY DISCUSSION

**Graphical representations of heterogenous, complex, patient groups**

In this complex, heterogeneous group of patients, there is a clear value to a network analysis, where differentiating between potential diagnoses is difficult. As an alternative approach to minimizing the complexity through simple modelling or univariate statistical analysis, it illuminates the clear, yet intricate, relationships between imaging, serological and CSF features in this cohort. This approach to embrace the remarkable diversity of this disease group aims to reveal probabilistic links previously not well characterized, wherein we reveal clear, directed relationships between specific features, both at the whole cohort level but also at the subgroup level, as a means for further evaluation in later research.

The two subgroups of CNS infection and hematological disease involvement can be difficult to distinguish in terms of univariate imaging features, whereas network construction allows for the demonstration of a wide array of connections, differing either in existence at all, or with regards to the probability of the features occurring together. Of note, the CNS infection network was larger, with more edges, than the disease recurrence counterpart, fitting with the broad spectrum of infectious disease, wherein the radiological appearances of intracranial infection vary immensely. The eigenvector and authority centrality of features between these groups should also be contrasted, to infer how imaging, serology and LP findings influence the overall composite disease picture differently. We affirm the importance of non-radiological tests, particularly LP and blood cultures for differentiating the two, but also use this network representation to illustrate specific imaging features with greater influence over the graphical structure of disease in the disease recurrence and CNS infection groups.

**SUPPLEMENTARY FIGURE LEGENDS**

**Supplementary Figure 1:** Consort diagram for study cohort.

**Figure 2:** **Graphical analysis of all patients with CNS involvement of hematological disease (n=109).** A) Schema for generation of the directed graphical network coalescing imaging, blood tests and lumbar puncture features, wherein features are treated as individual nodes. The conditional probability of one feature being present, given the other is present, is calculated for all possible edge combinations. B) Weighted adjacency matrices of these conditional probability networks, illustrating there exists clear visual difference between the CNS hematological disease involvement and infection groups. Color code by conditional probability value, wherein a value approximating 0 is dark colored whereas one approaching 1 is brighter/paler. C) Network representation of the entire patient cohort with all features. Nodes are color-coded according to their eigenvector centrality, and edge size and color is proportional to the directed conditional probability, with color key as shown. Abbreviations: CSF, cerebrospinal fluid; PCR polymerase chain reaction; WCC, white cell count.
